# Prognostic significance of GSTP1 in patients with triple negative breast cancer

**DOI:** 10.18632/oncotarget.19824

**Published:** 2017-08-02

**Authors:** Guanglei Chen, Hao Zhang, Lisha Sun, Yanlin Jiang, Zhen Xu, Huizi Gu, Hong Xu, Jie Yang, Yining Wang, Tiantian Xu, Yingchao zhang, Caigang Liu

**Affiliations:** ^1^ Department of Breast Surgery, Shengjing Hospital of China Medical University, Shenyang 110004, China; ^2^ Department of Surgical Oncology, The First Hospital of China Medical University, Shenyang 110013, China; ^3^ Department of Internal Neurology, The Second Hospital of Dalian Medical University, Dalian 116027, China; ^4^ Cancer Hospital of China Medical University, Shenyang 110042, China; ^5^ Liaoning Cancer Hospital & Institute, Shenyang 110042, China; ^6^ Department of Breast Surgery, The Second Hospital of JiLin University, Changchun 130041, China

**Keywords:** breast cancer, GSTP1, prognosis

## Abstract

**Background:**

Previous studies showed that glutathione S-transferase Pi 1 (GSTP1) is a critical metabolic driver that is heightened specifically in triple negative breast cancer (TNBC) and drives breast cancer pathogenicity. This study focuses on investigating the relationship between the expression of the GSTP1 protein and TNBC metastasis and prognosis in China.

**Results:**

Chi-square and Fisher's exact tests showed that tumor size (P=0.023) and clinical stage (P=0.049) were significantly associated with GSTP1 expression. Patients with high GSTP1 expression exhibited an improved survival rate compared with patients with low GSTP1 expression, but the difference was not statistically significant (P=0.437). On multivariate analysis, clinical stage proved to be an independent prognostic factor for survival in breast cancer.

**Materials and methods:**

A total of 175 patients with histologically confirmed TNBC, who also underwent radical surgery between January 2008 and November 2011 at the Liaoning Cancer Hospital, were enrolled. Immunohistochemistry was used to detect GSTP1 expression in breast cancer tissue from 175 patients. The correlations between GSTP1 expression and other parameters were evaluated using the Chi-square and Fisher's exact tests. Univariate and multivariate Cox regression analyses were performed to assess independent prognostic factors for survival. Associations of GSTP1 expression with clinical stage and prognosis were analyzed using Kaplan–Meier survival curves.

**Conclusions:**

Tumors with high GSTP1 protein expression were independently associated with low clinical stages in TNBC patients in China. The expression of the GSTP1 protein may be a novel prognosis marker for TNBC patients in China.

## INTRODUCTION

Breast cancer (BC) is one among the most common malignancies in women, accounting for about 23% of all newly diagnosed cancers and 14% of cancer-related deaths [1]. Although the incidence of breast cancer in China is lower than in the United States [2], studies have shown that the incidence of breast cancer has been rising in China [3]. Although there are several comprehensive treatment options, such as surgery, chemotherapy, and endocrine therapy, many patients still have high rates of metastasis and recurrence, which remain the primary cause of death in patients with breast cancer [4]. Patients with triple negative breast cancer (TNBC) account for about 15–20% of total BC cases, which have higher rates of metastasis and recurrence, and lower survival rates compared to other subtypes because these patients do not receive anti-receptor therapy. Therefore, other potential prognostic markers and new therapeutic targets for BC should be explored.

Human glutathione S-transferase (GST) consists of the Alpha, Mu, Pi, Omega, and Theta classes, a super family of dimeric phase-II metabolic enzymes that have an irreplaceable role in the cellular defense system [5, 6]. Louie S M. found that GST Pi 1 (GSTP1) was a new TNBC oncogene that governed the pathogenicity of cancer by regulating glycolysis, and energy and fat metabolism [7]. Although some reports had shown the association between GSTs and overall survival in BC patients, the results were not consistent [8–11]. Therefore, the goal of the present study was to investigate the relationship between the expression of the GSTP1 protein and the prognosis of patients with TNBC.

## RESULTS

### Relationships between GSTP1 expression and clinicopathological characteristics

**Figure 1:**
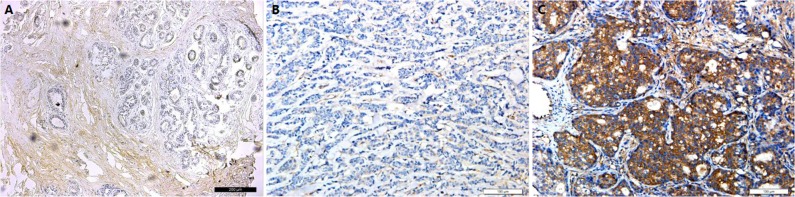
Immunohistochemical staining for GSTP1 **(A)** GSTP1 protein is absolutely negative in normal breast tissue. (Original magnification, 100× in A.) **(B)** GSTP1 protein showed negtive signals in triple-negative breast cancer tissues. **(C)** GSTP1 protein showed negtive signals in triple-negative breast cancer tissues. (Original magnification, 200× in B and C.)

The expression of the GSTP1 protein in 175 cases of breast tumors was obtained by IHC. Figure 1 shows the typical outcome of IHC staining. Of the 175 BC patients, approximately 77.1% of them had positive GSTP1 expression and 22.9% showed negative expression. We found that the positive rate of GSTP1 expression was significantly higher in smaller tumors (P=0.023, Table 1) after calculating the association between GSTP1 protein expression and clinicopathological data of breast tumors. In addition, the positive rate of GSTP1 protein expression was significantly different in clinical stages (CSs): the lower the CS of the tumor, the higher the positive expression rate of the GSTP1 protein (P=0.049, Table 1). However, there was no significant association between GSTP1 protein expression and the remaining clinicopathological characteristics, such as menopausal status (P=0.339), axillary lymph node status (P=0.071), Ki-67 status (P=0.936), pathological type (P=0.607), histological grade (P=0.750), and age (P=0.151) (Table 1). Thus, after univariate analysis, the variables significantly correlated with GSTP1 protein expression were tumor size and clinical stage.

**Table 1:** Association between GSTP1 expression and clinicopathological features in 175 patients with triple negative breast cancer

Variables	GSTP1 expression NO. (%)		P-value
	Negative	Positive	
No. of Patients	40 (100.0)	135 (100.0)	
Age (year)			0.151
≤45	7 (17.5)	39 (28.9)	
>45	33 (82.5)	96 (71.1)	
Menopausal status			0.339
Premenopausal	17 (42.5)	69 (51.1)	
Postmenopausal	23 (57.5)	66 (48.9)	
Clinical stage			0.049
1	3 (7.5)	32 (23.7)	
2	30 (75.0)	90 (66.7)	
3	7 (17.5)	13 (9.6)	
Tumor size			0.023
T≤2cm	4 (10.0)	42 (31.1)	
2cm<5cm≤	33 (82.5)	88 (65.2)	
T>5cm	3 (7.5)	5 (3.7)	
Pathologic type			0.607
Invasive dutal carcinoma	33 (82.5)	117 (86.7)	
Others	7 (17.5)	18 (13.3)	
Histological grade			0.750
1	0 (0)	1 (0.7)	
2	26 (65.0)	93 (68.9)	
3	14 (35.0)	41 (30.4)	
Ki-67 status			0.936
≤20	11 (27.5)	39 (28.1)	
>20	29 (72.5)	96 (71.9)	
ALNM			0.691
No	30 (75.0)	95 (70.4)	
Yes	10 (25.0)	40 (29.6)	

ALNM: axillary lymph node metastasis.

### Relationships between clinicopathological characteristics and prognosis

The average DFS was 91.489±2.258 months for all patients, 28 (16%) of whom developed recurrence or metastasis. Table 2 shows the results of the univariate analyses related to survival, including age, menopausal status, GSTP1 expression, tumor size, pathological type, histological grade, lymph node status, and clinical stage status. Univariate survival analysis showed that the CS (P<0.001) and lymph node status (P<0.001) were significantly correlated with DFS in BC patients (Table 2), which was consistent with many previous studies. With the help of Cox multivariate analysis comprised of variables examined with univariate analysis, we found that CS (HR 5.753, 95% CI: 1.963–16.865, P=0.001) was the only independent prognostic factor for DFS. The expression of GSTP1 protein expression had no significant influence on DFS based on uni- and multivariate Cox regression analyses (HR: 0.724, 95% CI: 0.319–1.645, P=0.441 and HR: 1.1461, 95% CI: 0.460–2.865, P=0.769, respectively). In both univariate and multivariate, patients with larger tumors had poor prognosis, but none of them were statistically significant (HR: 1.607, 95% CI: 0.773-3.341, P=0.204 and HR: 0.747, 95% CI: 0.319-1.749, P=0.501, respectively). In univariate analyses, the positive lymph nodes had a worse prognosis (HR 4.681, 95% CI: 2.187-10.019, P<0.001), whereas in multivariate analyses, lymph node status was not significantly associated with DFS, although the positive lymph node status had a poor prognosis (HR 1.524, 95% CI: 0.484-4.800, P<0.472). The remaining variables, including age, menopausal status, pathological type, histological grade,, were not discovered to be markedly relevant to DFS (Table 2).

**Table 2:** Univariate and multivariate analyses of clinicopathological risk factors for disease-free survival among breast cancer patients

Variable	DFS			
	Univariate analysis		Multivariate analysis	
	HR (95% CI)	P-value	HR (95% CI)	P-value
**Age**	1.320 (0.535-3.256)	0.546	2.100 (0.695-6.341)	0.188
**Menopausal status**	0.962 (0.459-2.018)	0.919	0.631 (0.254-1570)	0.322
**ALNM**	4.681 (2.187-10.019)	<0.001	1.524 (0.484-4.800)	0.472
**Tumor size**	1.607 (0.773-3.341)	0.204	0.747 (0.319-1.749)	0.501
**Pathological type**	1.266 (0.481-3.331)	0.633	0.905 (0.327-2.502)	0.848
**Histological grade**	1.444 (0.684-3.050)	0.335	1.014 (0.435-2.367)	0.974
**Clinical stage**	6.234 (3.147-12.350)	<0.001	5.753 (1.963-16.865)	0.001
**GSTP1 status**	0.724 (0.319-1.645)	0.441	1.146 (0.460-2.865)	0.769
**Ki-67 status**	1.193 (0.507-2.806)	0.687	1.411 (0.572-3.481)	0.454

HR: hazard ratio; CI: confidence interval.

### Survival

**Figure 2:**
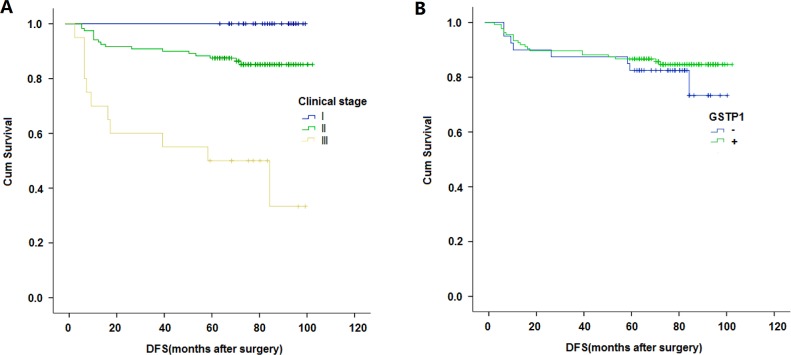
**(A)** Kaplan-Meier curves of disease-free survival (DFS) survival rates of triple-negative breast cancer patients based on clinical stage. **(B)** Kaplan-Meier curves of DFS survival rate of triple-negative breast cancer patients according to GSTP1 expression status.

The Kaplan–Meier survival curves correlated with GSTP1 expression and clinical stage are shown in Figure 2. When including GSTP1 protein expression as an influencing factor, patients with positive GSTP1 expression showed better DFS and patients with negtive GSTP1 expression showed poorer DFS, but this was not statistically significant (P=0.437). Consistent with many other studies, patients with clinical stage 1 were found to have better DFS when compared to those with clinical stage 2, and patients with stage 2 had a better DFS than patients with stage 3 tumors (P<0.001).

## DISCUSSION

Some previous studies have shown that there was a potential correlation between GSTP1 and BC, but there is no report on the relationship between GSTP1 protein expression obtained by IHC and prognosis in TNBC patients, particularly in China. We found that although some experts explored the relationship between GSTP1 and OS, the results obtained were inconsistent [8–11]. Although Louie S M. believed that GSTP1 [7], a new TNBC target, was a risk factor for BC and promoted BC, Song [14] reported that GSTP1 was not associated with BC risk by a meta-analysis of numerous case-control studies. However, Song [14] also found that GSTP1 can increase the risk of BC in Caucasian populations, while in Asian populations, GSTP1 and prognosis had no significant correlation [14, 15]. Therefore, our study was devoted to exploring the relationship between the expression of the GSTP1 protein obtained by IHC and the prognosis in TNBC patients.

In univariate analysis, patients with a low CS showed a higher positive GSTP1 protein expression rate, which was statistically significant (P=0.049). As demonstrated by the Kaplan–Meier survival curve, TNBC patients with positive GSTP1 expression displayed better DFS, and the DFS of patients with negative GSTP1 expression was poor, but there was no significant correlation between them (P=0.437). As is widely known, CS is directly related to the prognosis of BC patients, and has been widely demonstrated by many studies. However, uni- and multivariate Cox regression analyses showed that GSTP1 expression did not have a significant effect on prognosis. We considered that there are two reasons for this result; one is that the data we incorporated was unreasonable or untrustworthy and the other reason is that we had a relatively small sample size. To demonstrate the rationality of our data and to increase the credibility of the present study, we performed uni- and multivariate Cox regression analysis and constructed Kaplan–Meier survival curves for clinical staging and prognosis. As in many other studies, clinical staging is an independent prognostic factor in multivariate Cox regression, and the higher the clinical staging in the Kaplan–Meier survival curve, the lower the patient's DFS. Therefore, we believe the result that GSTP1 is significantly correlated to CS other than prognosis was due to our relatively small sample size in this study.

Univariate analysis showed that lymph node status is a factor for poor prognosis in BC, which is verified in many other studies. To our surprise, tumor size did not remarkably affect prognosis in our study because it is well known that tumor size, to a certain extent, also determines prognosis, which was also verified in many other studies. We think this might be due to the relatively small sample size.

The diagnosis and treatment of BC should be a rigorous process. With the continuous exploration of BC treatment models, a novel method of BC treatment used in clinical practice should be subject to scientific theory and experiment for support. Especially in different racial populations, extensive, scientific, and objective research is essential. Although many experts believe that GSTP1 is a risk factor for BC, we have found that GSTP1-positive TNBC patients have a better prognosis, whereas GSTP1-negative patients have poor prognosis in our study. In view of the above results, on the one hand, although it is desirable to continue a further study including larger numbers of samples, we have reason to believe that GSTP1 can serve as a prognostic factor for TNBC patients, which can provide important prognostic information for them. On the other hand, it might benefit treatment regimens and early diagnosis of BC.

Our research also has some limitations. First of all, it is obvious that our sample size is not enough. Second, our design was a single-center study, and the only included patients were from China. That is to say, if a more extensive sample of exploration in different racial groups was conducted, we might have received slightly different findings. Hence, further studies involving larger populations are required to verify these relationships.

In conclusion, our study revealed that there was a significant association between GSTP1 protein expression and CS in TNBC. GSTP1 expression analyzed by IHC may act as a predictive factor offering significant prognostic information for TNBC in patients in China.

## MATERIALS AND METHODS

### Patients and tumor samples

A total of 175 patients who were confirmed to have TNBC according to immunohistochemistry (IHC) results and had undergone mastectomy at the Liaoning Cancer Hospital between January 2008 and November 2011 were enrolled and retrospectively analyzed in this study. All patients were followed up by exchanging postal letters or via telephone interviews. Complete clinical records were evaluated. Estrogen receptor (ER), progesterone receptor (PR), HER2, and Ki-67 levels were analyzed using IHC. We obtained approval from the hospital's ethics committee and informed consent was obtained from each patient before surgery.

### Immunohistochemistry

GSTP1 expression was examined using IHC with rabbit anti-human GSTP1 polyclonal antibody (Proteintech, USA) at a dilution of 1:800, according to the method previously described for ER, PR, and Ki-67 with the following modifications: antigen retrieval was accomplished by incubation at 100°C in citrate buffer (pH 6.0) for 2 min and tumor specimens were treated with a peroxidase-conjugated secondary antibody, followed by incubation for 1 h at room temperature. The percentage of GSTP1-positive tumor cells was determined by manually counting the cytoplasmic-stained tumor cells. GSTP1 was considered positive when tumor cells stained were equal to or higher than 10%, because this cut-off value was often used in previous reports [12, 13] and was considered to be suitable for representing the biology of GSTP1-positive tumors. ER, PR, HER2, and Ki-67 expression or HER2 amplifications were examined by IHC. Cut-off values for ER, PR, and Ki67 were 10%, 10%, and 20%, respectively. The cut-off value for HER2 scoring was set at 3+ for IHC and 2.0 for FISH. The HER2 found to be 2+ for IHC was determined by means of the FISH classified as HER2-positive if the HER2 gene was found amplified.

### Statistical analysis

All data were analyzed with SPSS statistical software (version 17.0; SPSS Inc., Chicago, IL, USA). The correlation between GSTP1 expression and other parameters were evaluated with the Chi-square. Survival curves were estimated by the Kaplan–Meier method and compared with the log-rank test. Cox regression analysis (the enter method) for univariate and multivariate survival analysis was used to assess predictors related to disease-free survival (DFS), and the hazard ratios (HRs) and 95% confidence intervals (CIs) were calculated. All statistical analyses were two-sided and a P value of less than 0.05 was considered to be statistically significant.
